# Desire for labor companionship and its associated factors among pregnant women attending antenatal care at public health facilities in Debre Berhan City: a cross-sectional study

**DOI:** 10.3389/fgwh.2024.1426502

**Published:** 2025-01-07

**Authors:** Mulualem Silesh, Tesfanesh Lemma Demisse, Kidist Ayalew Abebe, Birhan Tsegaw Taye, Tebabere Moltot, Moges Sisay Chekole, Fetene Kasahun, Abebayehu Melesew Mekuriyaw, Tirusew Nigussie Kebede, Kibret Hailemeskel

**Affiliations:** ^1^ Department of Midwifery, Asrat Woldeyes Health Science Campus, Debre Berhan University, Debre Berhan, Ethiopia; ^2^ School of Medicine, Asrat Woldeyes Health Science Campus, Debre Berhan University, Debre Berhan, Ethiopia

**Keywords:** desire, labour companionship, pregnant women, antenatal care, Debre Berhan

## Abstract

**Background:**

Allowing women to have a companion of their choice during labor and delivery is a cost-effective strategy to enhance the quality of maternal care and promote a positive birth experience. Due to the limited studies on women's preferences for labour companionship, this study aimed to assess the desire for labour companionship and its associated factors among pregnant women attending ante-natal care at public health facilities in Debre Berhan City.

**Method:**

A facility-based cross-sectional study was conducted from August 1–30, 2022. A face-to-face questionnaire administered was used to collect data. Then, entered into Epi-Data version 4.6 and exported to SPSS version 25 for data analysis. In multivariable logistic regression analysis, variables with *P* < 0.05 with AOR and 95% CI were considered statistically significant.

**Result:**

Of 408 participants, 68.6% [95% CI: 63.8, 73.9] of pregnant women desired to have companionship in labour. Women who were living in urban [AOR: 2.32; 95% CI: 1.336, 4.022], had secondary level of education [AOR: 0.39; 95% CI: 0.207, 0.726], being pregnant for the first time [AOR: 1.88; 95% CI: 1.197, 2.945], women who had a good knowledge towards labour companion [AOR: 2.4; 95% CI: 1.522, 3.797] were statistically significant with desire on labour companionship.

**Conclusion:**

The magnitude of desire towards labour companionship in this study area was found high. Place of residence, educational attainment, number of pregnancies (gravidity), and level of knowledge about labour companions significantly contribute to women's desire for labour companionship. Therefore, to increase the desire for labor companions; antenatal education about its benefits should emphasized, particularly in rural areas and among less-educated communities. Providing tailored support for primigravida women and underserved populations can also help to integrate labor companionship into maternal care.

## Introduction

Improving the accessibility and availability of facility-based birthing has been a central goal in reducing maternal mortality and morbidity (1). Globally, an increasing number of births now occur in health facilities. However, the focus has shifted to enhancing the quality of care provided, as inadequate care remains a significant barrier to using facility-based childbirth services, particularly in low- and middle-income countries (2–4). Enhancing women's care experience is an integral part of ensuring better maternal and neonatal health outcomes (3).

Labor can be a challenging and intimidating experience for women. Labor companionship involves providing continuous support during labor and delivery, usually by someone the woman selects, such as a spouse, relative, friend, or trained doula (5). Having a labor companion improves the childbirth experience, fosters respectful care, empowers women's autonomy and decision-making, and contributes to better outcomes for mothers and newborns (3, 5–8). Evidence demonstrates that labor companionship has numerous benefits, including reduced labor duration, increased rates of spontaneous vaginal delivery, decreased need for cesarean sections and analgesia, and improved maternal satisfaction with the childbirth experience. Additionally, companions provide psychological support that alleviates fear, pain, and loneliness during labor (8–11). Despite its benefits, its uptake may vary due to cultural, social, institutional, and individual factors.

The World Health Organization (WHO) suggests having a chosen companion present for women during labor and childbirth, which is also highlighted in its Quality of Care Framework for maternal and newborn health, which emphasizes the importance of accessing emotional and social support according to women's preferences, considering it as core to the experience of care and to achieving positive person-centered health outcomes (7, 12).

Despite its benefits, its uptake may vary due to cultural, social, institutional, and individual factors. The Ethiopian Ministry of Health recommends allowing labor companionship as an integral component of compassionate and respectful care (13), but its utilization is limited. Women's desire for labor companionship is a crucial factor influencing its utilization (14–16). Understanding the preferences and desires of pregnant women for labor companionship, as well as the factors that influence their choices, is crucial for designing interventions that promote this essential aspect of care. However, evidence on the desire for labor companionship in Ethiopia is scarce. This study aims to assess the desire for labor companionship and identify its associated factors among pregnant women attending antenatal care at public health facilities in Debre Berhan City.

## Methods and materials

### Study design, setting, and period

A facility-based cross-sectional study was conducted at Debre Berhan City public health facilities, North Shoa Zone, Ethiopia from August 1–30, 2022. Debre Berhan is found in North Shoa Zone Amhara regional state 130 km from the capital city of Ethiopia, Addis Ababa. The city has five public health facilities; one Comprehensive Specialized referral hospital, one university hospital (Hakim Gizaw Hospital), and three health centers (Debre Berhan 04 Health center, Ayer Tena Health Center and Tebase Health Center). All these public health facilities provide ante-natal care (ANC) services. Finally, the study was done in all public health facilities of Debre Berhan City.

### Study population and eligibility criteria

All pregnant women who were attending ANC at public health facilities of Debre Berhan City were the study population. Women who attended ANC at public health facilities during the study period were included in the study, while pregnant women who were planned for elective cesarean section and critically ill were excluded.

### Sample size determination and sampling technique

The sample size for this study was calculated using a single population proportion formula $n=\frac{{\left(Z\alpha /2\right)}^{2}p(1-p)}{d^{2}}$ by considering the following assumptions: a 95% confidence level (1.96), and margin of error (5%), and the proportion of desire for birth companionship at Debre Markos city (57.45%). Also, by adding a 10% non-response rate, the final sample size was 413. Based on the average number of pregnant women attending ANC per month, the determined sample size was proportionally allocated to each public health facility. Study participants were chosen using a systematic random sampling technique. The calculated k^th^ interval was ~ 3; every three pregnant women attending ANC were included in each health facility. The first woman was chosen at random through the use of a lottery method.

### Data collection instruments and procedures

After receiving written informed consent from study participants, data were collected using a pre-tested structured interviewer-administered questionnaire. The questionnaire was prepared in English, translated into the local language (Amharic), and then back into English to preserve consistency. The questionnaire contains socio-demographic characteristics, reproductive and maternal health service use-related characteristics, knowledge about labour companionship, and the desire for labour companionship. Data were collected by five midwives under the guidance of a supervisor. Both the supervisor and data collectors underwent a one-day training session before the data collection period.

### Operational definitions

**Labour companionship**: Support provided by a partner, family member, or social network to laboring women by staining with her at all moments of the labor process (7).

**Desire for labour companionship**: refers to a woman's need to have a supporter of her choice during labour and childbirth and was measured by Yes/No by asking the question “Have you a desire for labor companionship while you are in labor?” (2, 17).

**Knowledge of companionship**: refers to a woman's awareness of their right to have a companion in labor and the benefits of companionship in labour. It was measured using 10-item questions and participants were asked to answer using “yes” and “No”. A value of 1 for correct and 0 for incorrect responses was given and the total score ranged from 0 to 10. Those women who scored ≥ mean were considered as having good knowledge and those who scored less than mean score were considered as having poor knowledge (17).

### Data quality control

The training was given to both supervisors and data collectors before the data collection period. Twenty-one pregnant women (5%) of the sample size, were employed to pre-test the questionnaire in the nearby Chacha health center. Every day, the supervisor checks the completeness and accuracy of each collected data.

### Data processing and analysis

The data were entered using Epi-Data version 4.6 and exported to SPSS version 25 for analysis. Binary logistic regression was done after generating descriptive statistics. In the bivariate logistic regression analysis, variables with a *P*-value less than 0.25 were entered into multivariable logistic regression analysis. The association of variables with desire towards labour companionship was declared using *P* < 0.05 and an AOR with 95% CI. Finally, text, tables, and graphs were used to present the results.

## Result

### Socio-demographic characteristics

A total of 408 pregnant women participated in this study, with a response rate of 98.8%. Of which, the participants' average age was 28.42 years old (SD ± 5.52), and 242 (59.3%) of participants were found between the ages of 25–34 years. Of the total respondents, 333 (81.6%) and 388 (95.1%) women were belonged to the Amhara ethnic group and currently married respectively (Table 1).

**Table 1:** Socio-demographic characteristics of women attending the ANC at public health facilities of Debre Berhan City, Ethiopia (*n* = 408).

Characteristics	Category	Frequency (*N*)	Percent (%)
Age (Years)	≤24	97	23.8
	25–34	242	59.3
	≥35	69	16.9
Residence	Urban	337	82.6
	Rural	71	17.4
Religion	Orthodox	311	76.2
	Protestant	52	12.7
	Muslim	45	11.1
Current Marital status	Married	388	95.1
	Unmarried★	20	4.9
Ethnicity	Amhara	333	81.6
	Oromo	61	15.0
	Tigrie	14	3.4
Educational level	No formal education	73	17.9
	Primary	125	30.6
	Secondary	97	23.8
	College and above	113	27.7
Occupational status	Housewife	174	42.6
	Employee	123	30.1
	Merchant	61	15.0
	Farmer	44	10.8
	Student	6	1.5
Husband's education level (*N* = 388)	No formal education	22	5.7
	Primary	72	18.6
	Secondary	96	24.7
	College and above	198	51.0
Family monthly income [in Ethiopian birr (ETB)]	<3,000	77	18.9
	3,000–4,999	104	25.5
	5,000–8,374	125	30.6
	≥8,375	102	25.0

★ Single, divorced and widowed.

### Obstetrics-related characteristics and knowledge of labour companionship

In the current study, 205 (50.2%) of the respondents were Primigravida and 360 (88.2%) of the participants' current pregnancies were planned. Also, this study revealed that; 45 (11%) and 29 (7.1%) of women had a history of obstetric complication and chronic illness respectively. Furthermore, 266 (65.2%) of pregnant women had a good knowledge of labour companionship (Table 2).

**Table 2:** Obstetric related characteristics and knowledge on labour companionship among women attending ANC at public health facilities of Debre Berhan City, Ethiopia (*n* = 408).

Characteristics	Category	Frequency (*N*)	Percent (%)
Gravidity	Primi-gravida	205	50.2
	Multigravida	203	49.8
Place of previous delivery (*N* = 210)	Home	10	4.8
	Public Health Center	115	54.8
	Private Clinic/Hospital	67	31.9
	Public Hospital	18	8.6
Ever had labour Companion in previous birth (*N* = 200)	Yes	61	30.5
	No	139	69.5
Ever informed about labour companionship by provider	Yes	143	35
	No	265	65
Pregnancy Status	Planned	360	88.2
	Unplanned	48	11.8
Number of ANC frequency	1	71	17.4
	2–3	235	57.6
	4 and above	102	25.0
Gestational age (in Trimester)	First Trimester	18	4.4
	Second Trimester	181	44.4
	Third Trimester	209	51.2
History of Obstetric complication in current Pregnancy	Yes	45	11.0
	No	363	89.0
Had history chronic diseases?	Yes	29	7.1
	No	379	92.9
Knowledge on Companionship	Poor Knowledge	142	34.8
	Good Knowledge	266	65.2

### Desire for labour companionship

**Figure 1:**
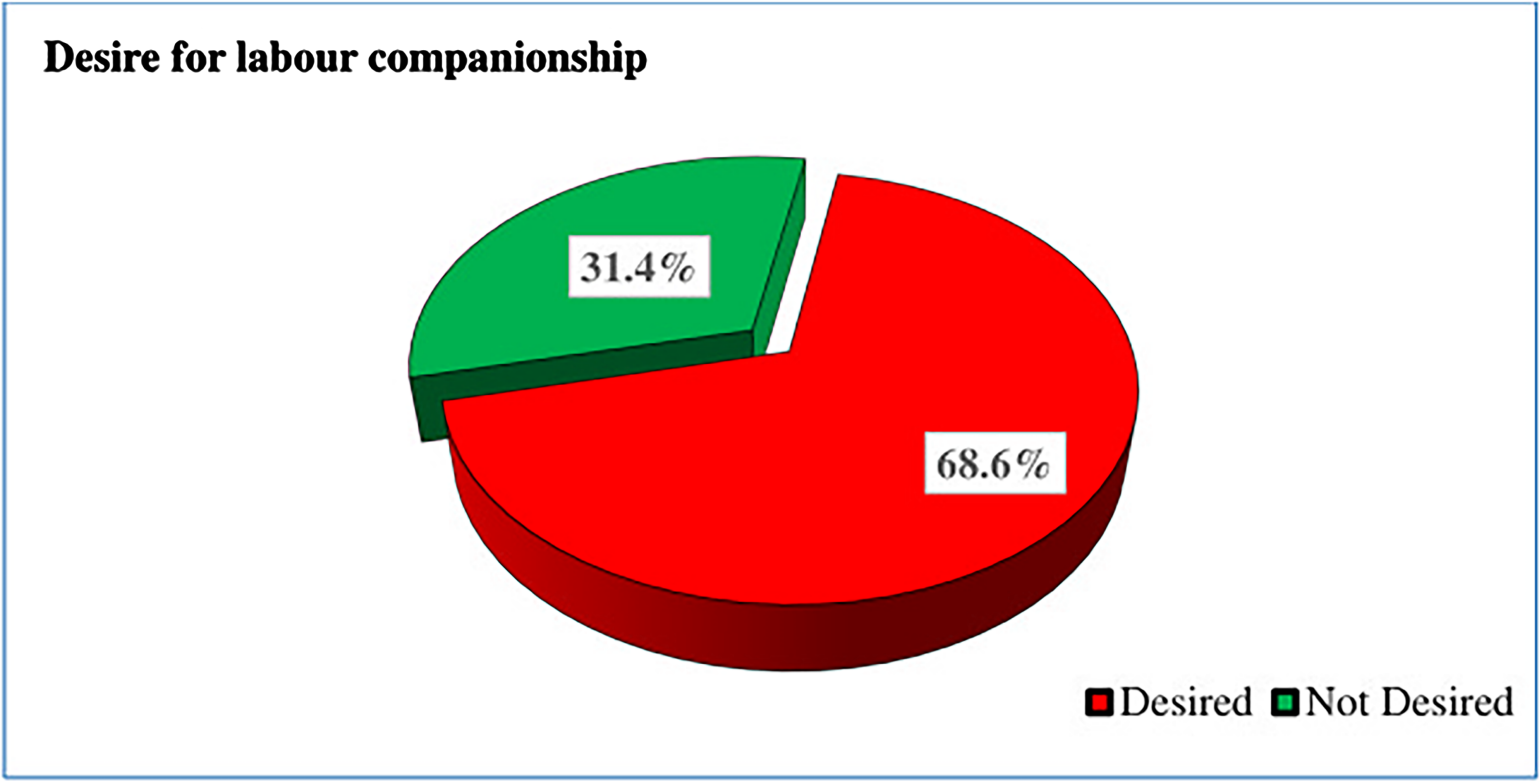
Desire for labour companionship among pregnant women attending ANC at public health facilities of Debre Berhan City, Ethiopia (*n* = 408).

Of the total respondents, 280 (68.6%) [95% CI: 63.8, 73.9] of pregnant women desire to have companionship in labour (Figure 1).

### Factors associated with the desire for labour companionship

In bivariable logistic regression analysis; maternal age, residence, occupational status, educational status, number of pregnancies (gravidity), pregnancy status, ever informed about labour companionship, having chronic diseases and knowledge of labour companionship were candidates for multivariable logistic regression analysis. But, in multivariable logistic regression analysis only maternal residence, educational status, number of pregnancies (gravidity), and knowledge towards labour companionship were statistically significant with desire towards labour companionship.

Women who were living in urban were 2.3 times more likely to desire to have a companion during labour than their counterparts [AOR: 2.32; 95% CI (1.336, 4.022)]. Pregnant women who had secondary-level education were 61% less likely to desire labour companions than women who had completed college and above [AOR: 0.39; 95% CI (0.207, 0.726)]. Women who were pregnant for the first time were almost 2 times more likely to desire to have labour companion compared to women who had been pregnant more than one time [AOR: 1.88; 95% CI (1.197, 2.945)]. Mothers who had a good knowledge towards labour companions were 2.4 times more likely to desire to have labour companions than their counterparts [AOR: 2.4; 95% CI (1.522, 3.797)] (Table 3).

**Table 3:** Factors associated with desire for labour companionship among women attending ANC at public health facilities of Debre Berhan City, Ethiopia (*n* = 408).

Variable	Desire for labour Companionship		COR, 95% CI	AOR, 95% CI	*P*-Value
	Yes *N* (%)	No *N* (%)			
Age in years					
≤24	61 (62.9)	36 (37.1)	0.55 (0.279, 1.099)	0.98 (0.430, 2.250)	0.969
25–34	167 (69.0)	75 (31.0)	0.73 (0.395, 1.342)	0.90 (0.435, 1.866)	0.779
≥35	52 (75.4)	17 (24.6)	1	1	
Residence					
Urban	242 (71.8)	95 (28.2)	2.21 (1.311, 3.733)	2.32 (1.336, 4.022)**	0.003
Rural	38 (53.5)	33 (46.5)	1	1	
Maternal occupational status					
Housewife	114 (65.5)	60 (34.5)	1	1	
Employee	83 (67.5)	40 (32.5)	1.09 (0.669, 1.783)	0.55 (0.286, 1.049)	0.069
Merchant	43 (70.5)	18 (29.5)	1.26 (0.668, 2.367)	0.99 (0.486, 2.038)	0.989
Farmer	37 (84.1)	7 (15.9)	2.78 (1.17, 6.615)	2.21 (0.821, 5.923)	0.116
Student	3 (50.0)	3 (50.0)	0.53 (0.103, 2.688)	1.28 (0.197, 8.257)	0.799
Maternal educational status					
No formal education	54 (74.0)	19 (26.0)	0.89 (0.453, 1.759)	0.76 (0.369, 1.547)	0.443
Primary	84 (67.2)	41 (32.8)	0.64 (0.363, 1.139)	0.70 (0.386, 1.276)	0.245
Secondary	56 (57.7)	41 (42.3)	0.43 (0.237, 0.774)	0.39 (0.207, 0.726)**	0.003
College and above	86 (76.1)	27 (23.9)	1	1	
Gravidity					
Primi-gravida	156 (76.1)	49 (23.9)	2.03 (1.323, 3.11)	1.88 (1.197, 2.945)**	0.006
Multigravida	124 (61.1)	79 (38.9)	1	1	
Pregnancy status					
Planned	252 (70.0)	108 (30.0)	1.67 (0.900, 3.087)	1.69 (0.870, 3.298)	0.121
Unplanned	28 (58.3)	20 (41.7)	1	1	
Ever informed about Labour Companionship by Provider					
Yes	105 (73.4)	38 (26.6)	1.42 (0.906, 2.228)	1.17 (0.718, 1.902)	0.530
No	175 (66.0)	90 (34.0)	1	1	
Had chronic diseases currently?					
Yes	23 (79.3)	6 (20.7)	1.82 (0.722, 4.584)	2.34 (0.874, 6.269)	0.091
No	257 (67.8)	122 (32.2)	1	1	
Knowledge towards labour companionship					
Poor	80 (56.3)	62 (43.7)	1	1	
Good	200 (75.2)	66 (24.8)	2.35 (1.523, 3.621)	2.4 (1.522, 3.797)**	0.000

** = Variables statistically significant at *P* < 0.05 **1** = reference category.

## Discussion

Women who had a desire for accompanied during labor and delivery were five times more likely to have a companion compared to those who did not (14). Exploring pregnant women's preferences for having birth companions during labor and delivery is crucial. This understanding helps ensure health facilities are ready to accommodate such companions and fosters positive attitudes among healthcare providers. Allowing women to have a chosen companion during childbirth is a low-cost, effective way to improve the quality of maternity care. According to this study, 68.6% [95% CI: 63.8, 73.9] of pregnant women desired to have a companion during labour which is lower than studies done in Nigeria (92%) (18, 19) Malawi (83.6%) (20), Kenya (81%) (21), and South Wollo, Ethiopia (87.3%) (17). However, this finding was higher than the previous studies conducted in Debre Markos, Ethiopia (57.45%) (2). The observed discrepancy could be attributed to variations in socio-cultural and socio-demographic characteristics, as well as differences in the timing of the studies. For instance, cultural norms in countries like Nigeria, Malawi, and Kenya may strongly support the presence of a labor companion, leading to a higher desire for it. Additionally, in the current study, the majority of participants (82.6%) were urban residents, compared to only 35.5% in the Debre Markos study. Urban women generally have greater access to education and healthcare information, which increases their awareness of the benefits of having a labor companion.

Women who were residing in urban were 2.3 times more likely to desire to have a companion during labour than womenwho residein rural area. This might be due to the increased exposure to modern healthcare practices, better access to antenatal care services, and greater awareness of the benefits of labour companions through media or healthcare systems, which could shape their preferences. Additionally, cultural differences between urban and rural areas might influence women's perceptions of support during labour. However, this finding contradicts with the study conducted in South Wollo, Ethiopia (17). The possible variation might be, in South Wollo, the higher participation of rural women in the study may have influenced the result.

Pregnant women with a secondary-level of education were 61% less likely to desire labour companion compared to those women who had completed college and above. This finding is supported by the studies conducted in Ghana (22) and Nigeria (19). Also, a study finding in Ethiopia found that pregnant women who had a diploma and higher educational level had good knowledge towards labour companionship than women who had no formal education (17). This could be due to higher education may enhance awareness and understanding of the role and importance of labour companionship, leading to increased preferences among educated women.

Women who were pregnant for the first time were nearly twice as likely to desire labour companions compared to women who had been pregnant before. The finding of this study showed that primiparous (delivered for the first time) were two times more likely to be accompanied by their labor companion during childbirth in the health facilities than those women who were multiparous. This might be due to this finding aligns with women who were pregnant for the first time may experience greater anxiety and uncertainty about the labour process and perceived that the presence of a companion could provide emotional, informational, and physical support, making the experience less daunting.

Mothers who had a good knowledge of labour companions were 2.4 times more likely to desire to have labour companions than their counterparts. This can be explained by the fact that women with good knowledge about labour companionship could have a higher chance of understanding the potential benefits of labour companionship during labour and delivery, and likely influences attitudes. Therefore, their knowledge may help to functionally contribute to having a desire for it.

### Strengths and limitations of the study

The study provides valuable insights into pregnant women's desire for labour companionship in a specific Ethiopian context, contributing to limited existing literature on this topic. However, as a cross-sectional study, it cannot establish causality between the identified factors and the desire for companionship. Additionally, since the study was conducted in public health facilities in a single city, the findings may not be generalizable to other regions with differing cultural or healthcare contexts. Lastly, the reliance on self-reported data may introduce social desirability biases.

## Conclusion

The study revealed a high level of desire for labour companionship among pregnant women in the area. Place of residence, educational attainment, number of pregnancies (gravidity), and level of knowledge about labour companion are significantly contribute to women's desire for labour companionship. Therefore, to increase the desire for labor companionship, antenatal education should emphasize its benefits, particularly in rural areas and among less-educated communities. Providing tailored support for primigravida women and underserved populations can also help to integrate labor companionship into maternal care. Additionally, the authors recommend conducting larger multicenter studies using a mixed-methods approach to explore the role of health system and service provider factors.

## Data Availability

The original contributions presented in the study are included in the article/Supplementary Material, further inquiries can be directed to the corresponding author.
